# Sympathetic ^123^I-metaiodobenzylguanidine index for Lewy body disease: probability-based diagnosis and identifying patients exempt from late imaging

**DOI:** 10.1007/s12149-024-01950-4

**Published:** 2024-06-13

**Authors:** Kenichi Nakajima, Takeshi Matsumura, Junji Komatsu, Hiroshi Wakabayashi, Kenjiro Ono, Seigo Kinuya

**Affiliations:** 1 Department of Functional Imaging and Artificial Intelligence, Kanazawa University, 13-1 Takara-machi, Kanazawa, 920-8640 Japan; 2 Department of Nuclear Medicine, Kanazawa University, Kanazawa, Japan; 3 Department of Neurology, Kanazawa University Graduate School of Medical Sciences, Kanazawa, Japan

**Keywords:** Sympathetic imaging, Neurodegenerative disease, Logistic model, Quantitation

## Abstract

**Objective:**

We aimed to establish a practical diagnostic index for Lewy body diseases (LBD), such as Parkinson’s disease and dementia, with Lewy bodies in outpatient settings and criteria for exempting patients from late imaging.

**Methods:**

We acquired early and late ^123^I-metaiodobenzylguanidine (MIBG) images from 108 consecutive patients with suspected LBD and standardized heart-to-mediastinum (H/M) ratios for collimator conditions. Exclusions included young-onset Parkinson’s disease (age < 50 years) and genetic transthyretin-type amyloidosis. We developed logistic models incorporating H/M ratios with or without age (*n* = 92). The sympathetic MIBG index for LBD (SMILe index), categorized LBD likelihood from 0 (lowest) to 1 (highest). Diagnostic accuracy was assessed as the area under the receiver operating characteristic (ROC) curve (AUC). The characteristics of the new index were compared with H/M ratios. The need for late imaging was explored using the SMILe index.

**Results:**

Early or late SMILe indexes using a single H/M ratio variable discriminated LBD from non-LBD. The AUC values for early and late SMILe indexes were 0.880 and 0.894 (*p* < 0.0001 for both), identical to those for early and late H/M ratios. The sensitivity and the specificity of early SMILe indexes with a 0.5 threshold were 76% and 90%, achieving accuracy of accuracy 86%. Similarly, the late SMILe index demonstrated a sensitivity of 76% and specificity of 87%, with an accuracy of 84%. Early SMILe indexes < 0.3 or > 0.7 (representing 84% patients) indicated a diagnosis without a late MIBG study.

**Conclusion:**

The ^123^I-MIBG-derived SMILe indexes provide likelihood of LBD, and those with a 50% threshold demonstrated optimal diagnostic accuracy for LBD. The index values of either < 0.3 or > 0.7 accurately selected patients who do not need late imaging.

**Supplementary Information:**

The online version contains supplementary material available at 10.1007/s12149-024-01950-4.

## Introduction

The clinical application of ^123^I-metaiodobenzylguanidine (^123^I-MIBG) as a radiopharmaceutical for sympathetic nerve imaging was approved in Japan during 1992 [1]. Predominantly used to determine ischemic heart diseases and heart failure, ^123^I-MIBG is now recognized as a Class IIa indication (weight of evidence/opinion in favor of usefulness/effectiveness) in the guidelines of the Japan Circulation Society [2]. Research into neurology applications during the early 2000s established substantially reduced ^123^I-MIBG uptake even at the early stages of Parkinson’s disease (PD) [3–6]. Clinical diagnostic criteria for PD were established by the Movement Disorder Society (MDS) for clinical research applications and to assist clinical diagnoses [7, 8]. Hence, ^123^I-MIBG is now included as a supportive criterion within this guideline. Subsequent applications to dementia with Lewy bodies (DLB) [9–11] have discovered markedly diminished sympathetic MIBG activity in the heart. A Japanese multicenter study of DLB and Alzheimer disease (AD) has confirmed a robust role of ^123^I-MIBG in the differentiation of patients with dementia [4, 12].

Against this backdrop, two-thirds of ^123^I-MIBG studies are used to diagnose patients with neurodegenerative diseases, particularly Lewy body diseases (LBD) including PD, DLB, and pure autonomic failure. Recognized as a pivotal imaging diagnostic method for decision-making regarding LBD, ^123^I-MIBG was incorporated into the Japanese practice guidelines 2018 for PD [13]. It was also acknowledged as an imaging biomarker for Lewy body-related diseases [14], at the fourth consensus report of the DLB Consortium [15, 16]. The Ministry of Health, Labor and Welfare in Japan officially approved the application of ^123^I-MIBG to neurological diseases in 2023, specifically for cardiac scintigraphy to diagnose PD and DLB. That late images acquired 3‒4 h after ^123^I-MIBG administration could be omitted should be carefully determined. This is understood based on the results of early images, patient's condition, and risk/benefit analysis. However, criteria for deciding which patients could avoid late imaging have not been defined.

The heart-to-mediastinum (H/M) ratio derived from planar ^123^I-MIBG images is commonly used for diagnosis, and the washout rate (WR) can be simultaneously calculated [17]. We have previously focused on standardizing differences in collimators and cameras, which are key factors contributing to variations in H/M ratios across facilities [18, 19]. This standardization has been embraced for clinical use in Japan [20], Europe [21], and Taiwan [22], and has enhanced the reliability of the diagnostic index.

The primary objective of the present study was to create a user-friendly model-based diagnostic index that would be suitable for application in clinics for diagnosing LBD. We also aimed to establish practical criteria for omitting the need for late images after early images have been acquired.

## Methods

### Patients

**Fig. 1:**
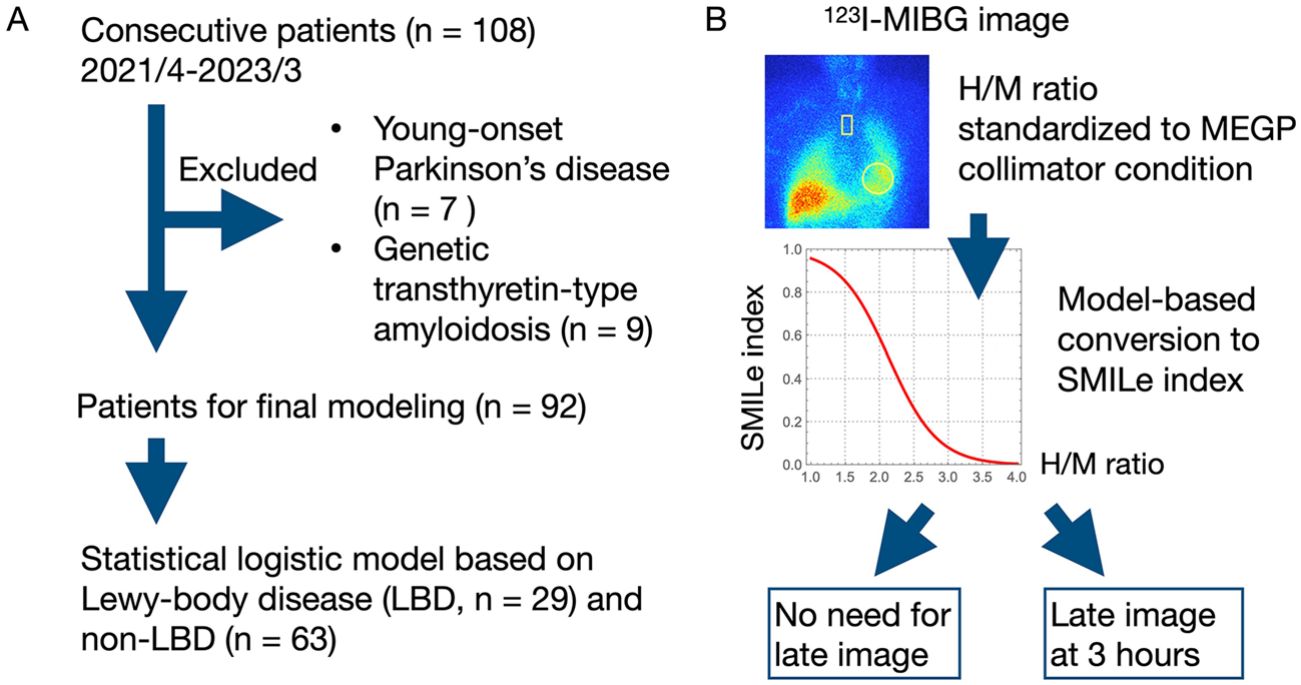
Selection of patients to create a diagnostic model **(A)** and steps for using the SMILe index **(B)**. *H/M* heart to mediastinum; *MEGP* medium-energy general purpose; *MIBG* metaiodobenzylguanidine; *SMILe* sympathetic MIBG index for Lewy body disease

Over a three-year period spanning from April 2021 to March 2023, we examined 108 consecutive patients referred for neurological diseases who participated in a ^123^I-MIBG study (Fig. 1). The mean age was 69 ± 11 (32‒90) years and males accounted for 57%. Seven of the patients were diagnosed with young-onset and/or familial Parkinson’s disease (mean age, 50 ± 18 years; onset 31‒47 years), and nine were diagnosed with genetic transthyretin-type amyloidosis (mean age, 64 ± 12 years).

Since an initial statistical analysis revealed early and late H/M ratios of 3.0 ± 0.5 and 3.2 ± 0.6 for young-onset PD, these patients were excluded from creating the diagnostic model. The exclusion from the model was due to their distinct biomarker profiles and genetic PD variants with a non-α-synuclein etiology, which affect the ^123^I-MIBG H/M ratio depending on the type of mutation [14, 23–25]. The variation in H/M ratios for amyloidosis was similarly wide (early vs. late: 2.1 ± 0.7 vs. 2.0 ± 1.0). Since the ^123^I-MIBG study in amyloidosis was not intended to differentiate between LBD and non-LBD, these patients were also excluded. Table 1 summarizes the baseline ^123^I-MIBG data. The diagnostic model was constructed using the remaining 92 patients focusing on a differential diagnosis of LBD. A diagnosis of PD has been independently established using the MDS clinical diagnostic criteria 2015 [7]. Diagnoses of DLB adhered to the DLB Consortium criteria [15]. The patients with LBD (*n* = 29) consisted of 17, seven, and five, with clinically established PD, probable PD, and probable DLB, respectively.

**Table 1:** Baseline demographics of consecutive patients (*n* = 108)

	Parkinson’s disease	Dementia with Lewy bodies	Young-onset Parkinson’s disease*	Genetic TTR amyloidosis*	Non-Lewy diseases
Patients (n)	24	5	7	9	63
Male/Female (n)	15/9	3/2	4/3	7/2	28/35
Age (y, range)	71 ± 10 (53 − 86)	74 ± 7 (67 − 85)	50 ± 18 (32 − 81)^†^	64 ± 12 (44 − 74)	70 ± 8 (51 − 90)
Early H/M ratio	1.9 ± 0.6	2.0 ± 0.8	3.0 ± 0.5	2.1 ± 0.7	2.9 ± 0.6
Late H/M ratio	1.7 ± 0.6	1.7 ± 0.8	3.2 ± 0.6	2.0 ± 1.0	3.0 ± 0.8
Washout rate (%)	54 ± 24	55 ± 24	16 ± 113	46 ± 30	21 ± 18

*Excluded from this study. ^†^Age at onset, 31 − 47 years. *H/M* heart-to-mediastinum; *TTR* transthyretin

Non-Lewy body-related diseases (*n* = 63) within the cohort comprised progressive supranuclear palsy (*n* = 19), multiple system atrophy (*n* = 12), corticobasal syndrome (*n* = 5), drug-induced (*n* = 2), cerebrovascular (*n* = 2), and non-specified (*n* = 8) parkinsonism. The 15 remaining patients were diagnosed with normal pressure hydrocephalus, peripheral neuropathy, motor neuron disease including amyotrophic lateral sclerosis, alcoholic neuropathy, spinal cord disease, and psychological disorders. Complications included type 2 diabetes mellitus (*n* = 17), coronary artery disease (*n* = 2), atrial fibrillation (*n* = 3), and chronic heart failure (*n* = 1).

### ^123^I-MIBG study

Scintigraphic images were obtained at 20 and 180 min after intravenously injecting111 MBq of ^123^I-MIBG into patients. Early and late images were acquired using anterior planar and single-photon emission computed tomography (SPECT) imaging. We analyzed only planar anterior images, although SPECT images were routinely used for image interpretation. The planar images were acquired under the parameters of 256 × 256 matrices, acquisition for 500 s, and energy for ^123^I centered at 159 keV with a 20% window.

Average heart and mediastinum values were calculated using the semi-automatic software smartMIBG that we developed (PDRadiopharma, Inc., Tokyo, Japan) [26]. Regions of interest were defined as circular for the heart and rectangular (10% of the width and 30% of the height) for the upper mediastinum. The H/M ratio was standardized with a low-medium energy collimator (conversion factor, 0.83) to a medium energy collimator condition (conversion factor, 0.88) as described [19, 20]. Washout rates (WRs, %) were calculated using early and late average heart counts as:

(early−late counts × correction factor for decay)/early count × 100,

after background correction with the mediastinum count, and decay was corrected using the formula:

1/0.5^(time [h] /13 [h]) [27].

We also calculated the H/M ratio-based WR (%) as (early H/M−late H/M) × 100/early H/M [28].

Creation of the diagnostic model.

We used a statistical logistic model to estimate LBD, including PD and DLB. Early and late H/M ratios and age emerged as significant variables for diagnosing LBD from the initial model that included all patients (*n* = 108). The unit odds ratios were 7.44 (*p* < 0.0001) for the late H/M ratio and 1.06 for age (*p* = 0.024) in the multivariable logistic model. However, due to deviation in the results caused by young-onset PD patients having almost normal H/M ratios, we decided to use 92 patients for calculating early and late H/M ratios with or without an age factor.

The logistic model-based sympathetic MIBG Index for Lewy body disease (SMILe) was formulated using the equation:

Probability = 1/(1 + Exp [−(intercept + Σ estimate × variable)]),

which corresponds to the likelihood of LBD ranging from 0 to 1 (the lowest to the highest probability).

We used additional statistical data from a Japanese multicenter study to assess the ability of the model to discriminate DLB from AD [12]. The final diagnoses were established over a 3-year follow-up. The statistical data table did not contain any personal information, and only H/M ratios and final definitive diagnosis were used for validation.

### Statistics

The data are presented as means with standard deviation (SD). Differences in variables between groups were assessed using T tests and analyses of variance. Early and late indexes were compared using paired t tests. The logistic model was constructed using H/M ratios and/or WR with and without an age factor. Early and late indexes were compared using linear regression analysis. The area under the receiver operating characteristic (ROC) curve (AUC) was calculated. We assessed the need for late images by the categorizing SMILe indexes of < 0.3, 0.3 − 0.7, and > 0.7 as a low, borderline, and high likelihood of LBD, respectively. We also evaluated the characteristics of H/M ratios and SMILe indexes, using histograms, as well as 2-dimensional (2D) density plots that showed a probability density function with 2D coordinates. All data were analyzed using JMP pro software v. 17.0 (SAS Institute Inc., Cary, NC, USA). Values with *P* < 0.05 were considered significant. We used Mathematica v. 14.0 software (Wolfram Research Inc., Champaign, IL. USA) for modeling and simulations.

## Results

### H/M ratio and WR for LBD and non-LBD

**Fig. 2:**
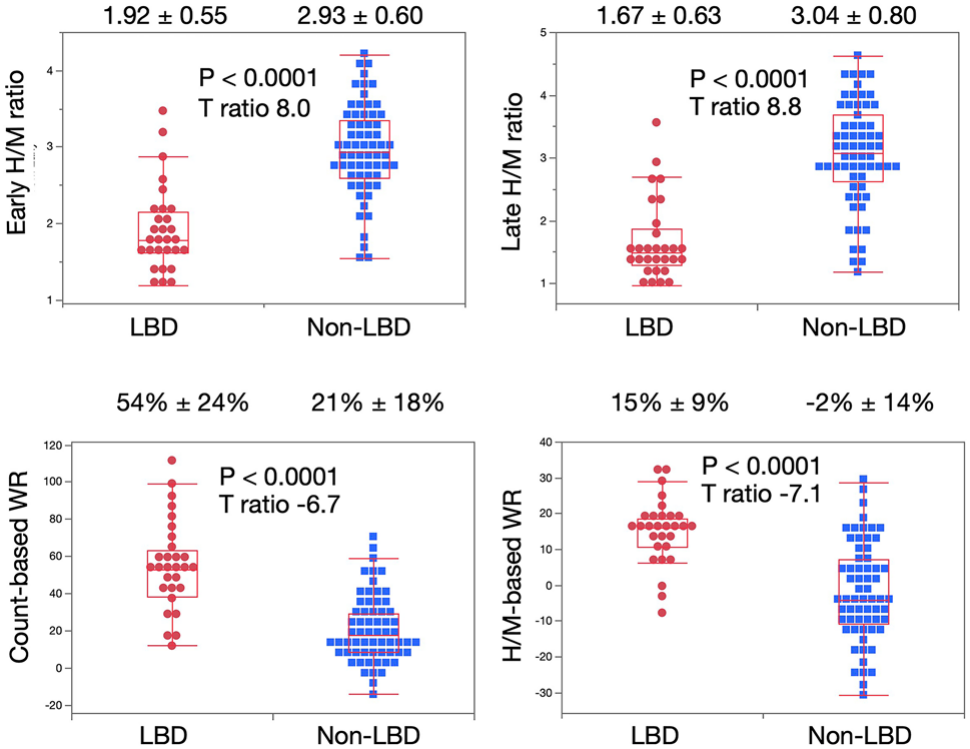
Comparison of diagnostic indexes between patients with and without LBD. Means ± SDs are shown above each chart. *H/M* heart-to-mediastinum; *LBD* Lewy body disease; *SDs*, standard deviations; *WR*, washout rate

Early and late H/M ratios and WRs were compared between groups with and without LBD (Fig. 2). All variables significantly differed (*p* < 0.0001) between the groups. The late H/M ratio was significantly lower than the early H/M ratio in the LBD group (1.67 ± 0.63 vs. 1.92 ± 0.55, *p* < 0.0001; paired *t* tests).

### Logistic model and SMILe index

**Fig. 3:**
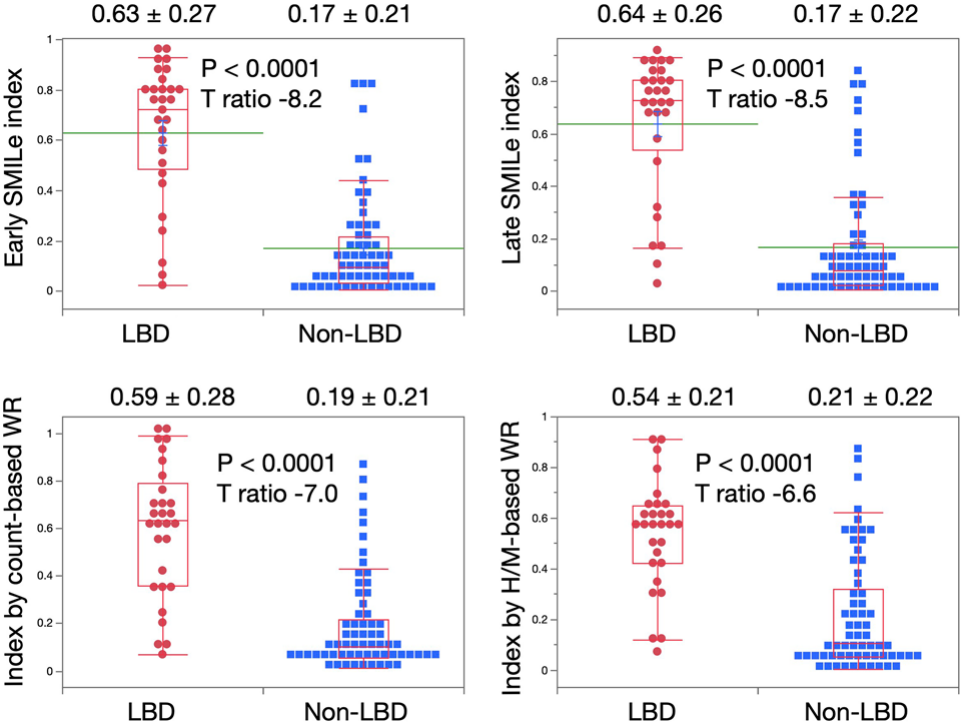
Early and late SMILe indexes for patients with and without LBD. Lower panels, indexes generated using count- and H/M-based WRs. Means and SDs are shown above each chart. *H/M* heart-to-mediastinum; *SDs* standard deviations; *WR* washout rate

**Fig. 4:**
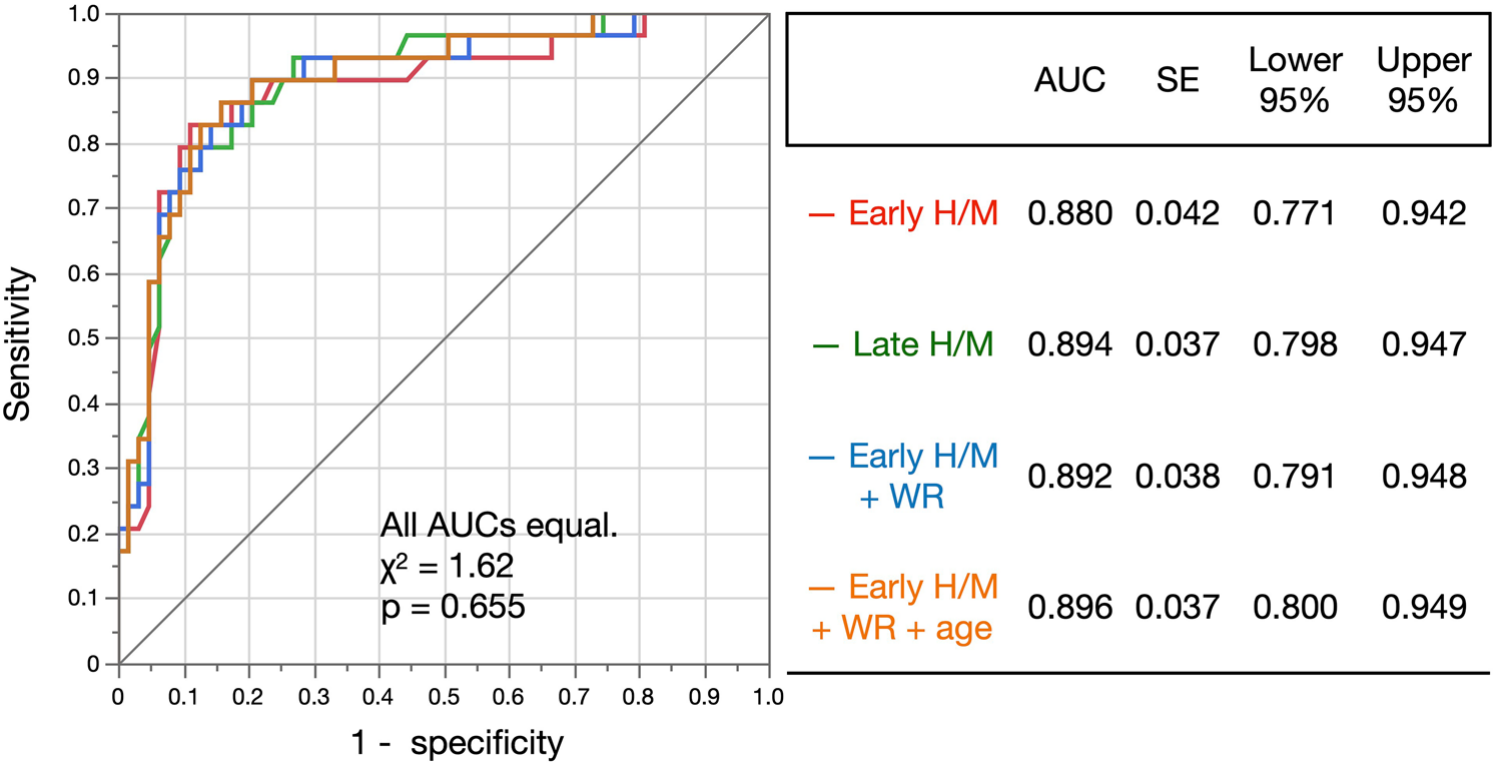
Receiver operating characteristic curves and area under the curve for discriminating Lewy body disease. Models with early and late H/M ratios (red and green, respectively). Comparison of WR (blue) and WR combined with age (orange) were compared. *AUC* area under the ROC curve; *H/M* heart-to-mediastinum; *ROC* receiver operating characteristics; *WR*, washout rate

The parameter estimates and the intercepts were 2.79 and −5.93, and 2.18 and −4.23, for the early and late H/M ratios, respectively (Table 2). Figure 3 shows that the early and late values, when SMILe indexes were applied, were 0.63 ± 0.27 vs. 0.17 ± 0.21 and 0.64 ± 0.26 vs. 0.17 ± 0.22, respectively, for the groups with and without LBD (*p* < 0.0001). The AUCs of early H/M combined with WRs were comparable (0.892) as shown in Fig. 4. Due to slight age dependence on H/M ratio in Japanese normal MIBG databases [29], we also tested a model incorporating early H/M ratios, WRs and age. However, the AUC remained comparable at 0.896. Overall, since statistically significant differences were not achieved compared with the model with only the early H/M ratio, we opted for a logistic model using a single H/M ratio.

**Table 2:** Logistic model analysis of early and late heart-to-mediastinum ratios

Term	Parameter estimate	SE	χ^2^	*P* > χ^2^	Unit odds ratio
Early					
H/M ratio	− 2.79	0.57	24.23	< 0.0001	16.29
Intercept	5.93	1.32	20.05	< 0.0001	
Late					
H/M ratio	− 2.18	0.43	25.57	< 0.0001	8.88
Intercept	4.23	0.95	19.92	< 0.0001	

*H/M* heart-to-mediastinum; *SE* standard error

The AUCs were 0.880 (*p* < 0.0001) and 0.894 (*p* < 0.0001) for early and late SMILe indexes, respectively. The sensitivity and specificity were 76% and 90% with an accuracy of 86% for early SMILe index, and 76% and 87% with an accuracy of 84% for late SMILe index at a threshold of 0.5. Since the SMILe indexes are based on a logistic function relationship with H/M ratios, the AUCs for the early and late H/M ratios were identical to those of SMILe index (0.880, *p* < 0.0001 and 0.894, *p* < 0.0001, respectively). The summed values of sensitivity and specificity were the highest at a threshold of 0.43 for the early SMILe index (sensitivity 83%, specificity 89%, accuracy 87%) and 0.49 for late SMILe index (sensitivity 79%, specificity 87%, accuracy 85%).

### Relationship between early and late H/M ratio and SMILe index

**Fig. 5:**
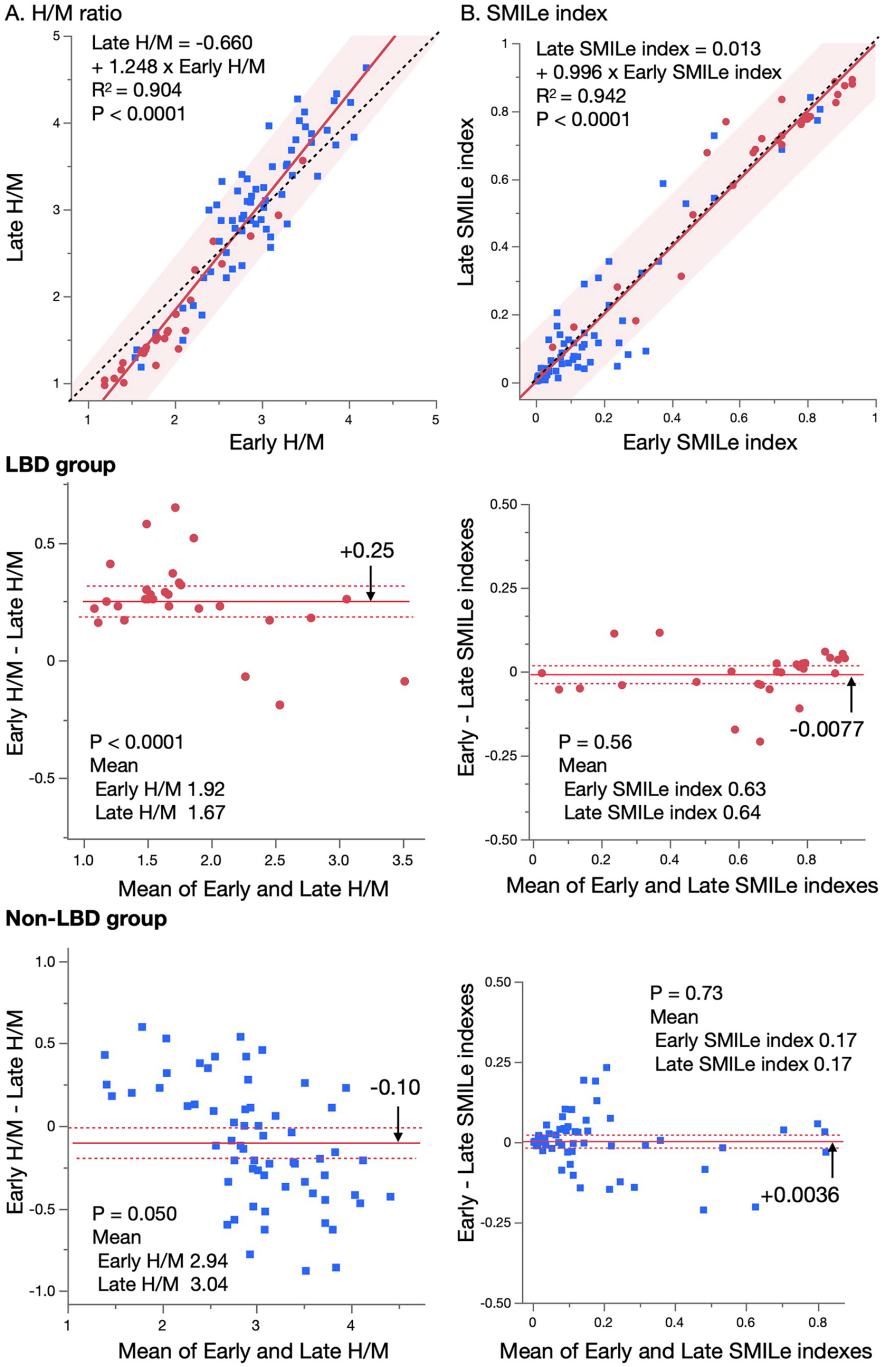
Relationship between early and late heart-to-mediastinum (H/M) ratios **(A)** and sympathetic index for Lewy body disease (SMILe index) **(B)**. Red line and shaded area, linear regression and confidence interval, respectively. Dashed line, identity. Mid- and lower panels, Brand–Altman plot shows means vs. difference

Figure 5 shows a positive correlation when a relationship between early and late H/M ratios was evident (*p* < 0.0001). However, the H/M ratio was lower in the late image (mean difference 0.25, *p* < 0.0001). Patients without LBD who had lower and higher H/M ratios had positive and negative differences respectively (early minus late H/M), indicating proportional errors. In contrast, the SMILe index aligned both early and late indexes along the identity line (intercept 0.013, slope 0.996). A Brand–Altman plot confirmed the absence of fixed and proportional errors.

**Fig. 6:**
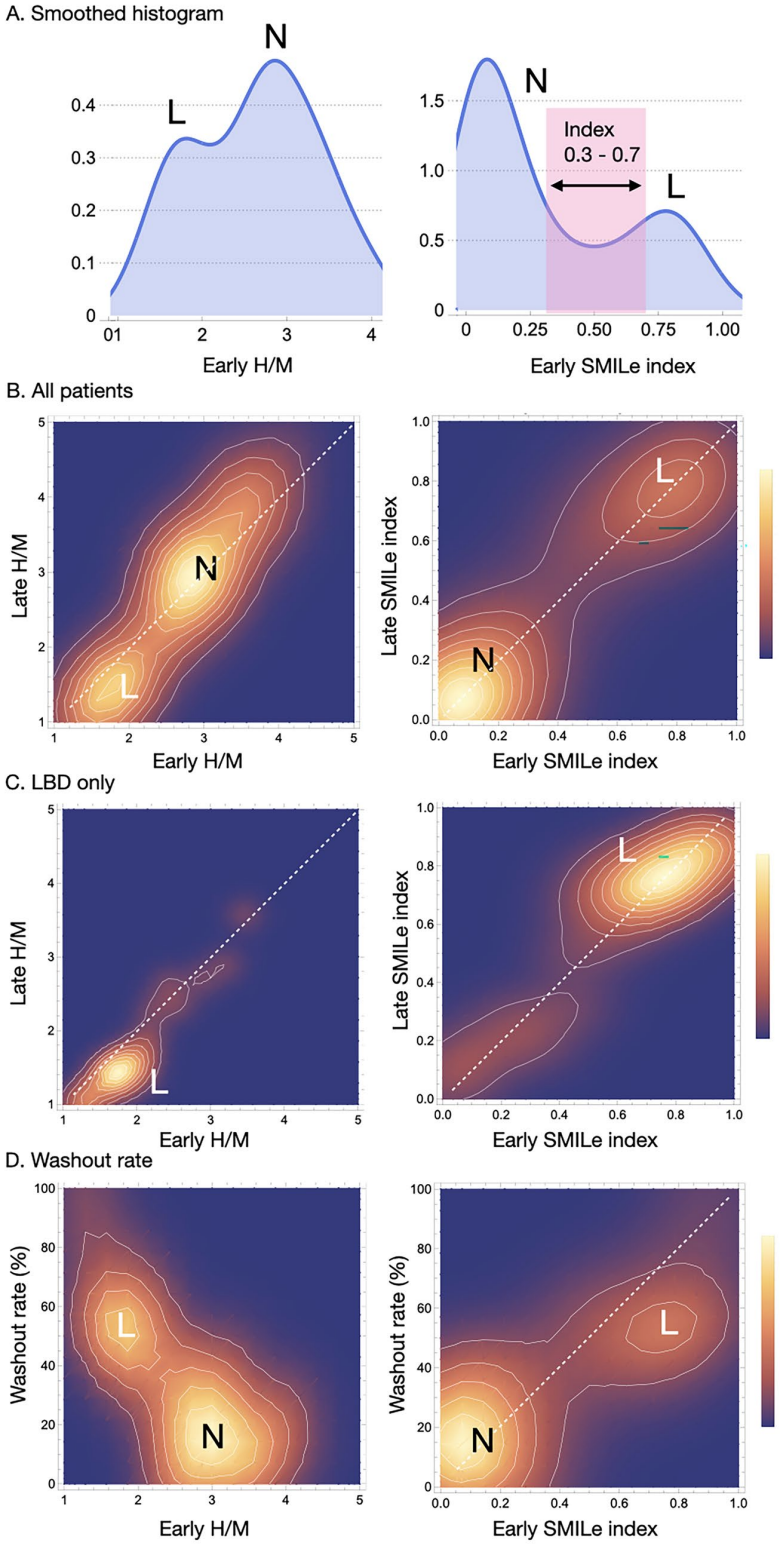
Histogram and 2D density plot of early H/M ratio and SMILe index. A: Left, histogram patterns smoothed for data density; right, SMILe index; red column, early SMILe index between 0.3 and 0.7. B: Distribution density map compares early and late parameters. C: Patients with only LBD. D: Relationships between WR and early H/M ratio (left) and SMILe index (right). The maximum value per display scale is 100%. L and N, with and without LBD, respectively. Dashed line, identity. *2D* 2-dimensional; *LBD* Lewy body disease; *SMILe* sympathetic index for Lewy body disease; *WR* washout rate

Histogram and histogram density plots revealed a distinct relationship among early and late SMILe indexes, H/M ratios, and WR (Fig. 6). The histogram was smoothed to delineate global peaks for density patterns (Panel A). The distributions of early H/M ratios and SMILe indexes were bimodal, but those of the latter were clearer with and without LBD. The relationship between early and late SMILe indexes also offered better separation than the plotted H/M ratios (Panels B and C). The early SMILe index and WR were obviously higher in the group with, than without LBD (Panel D).

### Requirement for late H/M ratios

**Fig. 7:**
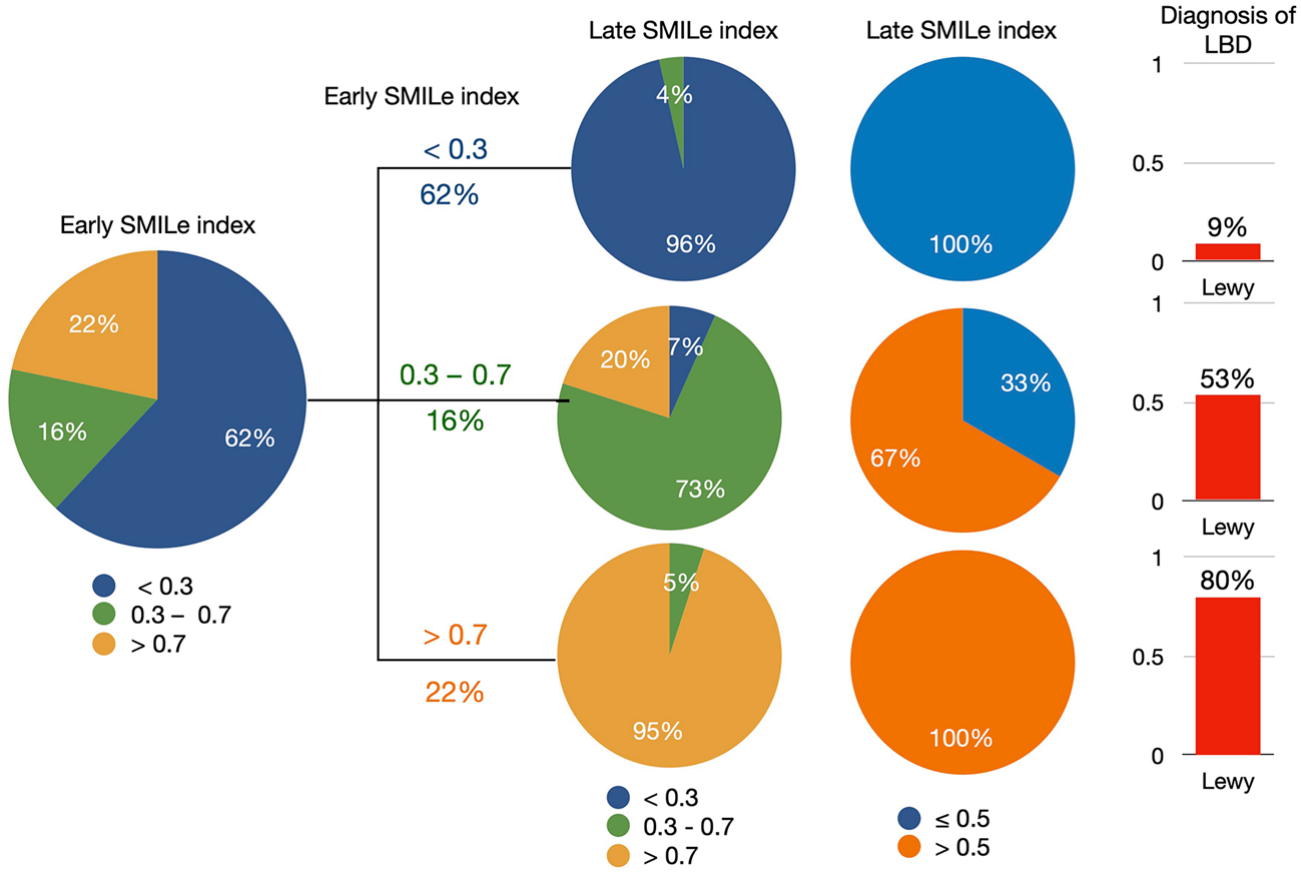
Changes in LBD diagnoses between early and late SMILe indexes. The SMILe index was classified as < 0.3 (low), 0.3 − 0.7 (intermediate), and > 0.7 (high) likelihood of LBD, and as ≤ 0.5 and > 0.5 (LBD negative and positive, respectively). Right bar, proportions of final LBD diagnosis. *LBD* Lewy body disease

Diagnostic categories did not change in 96% and 95% of patients with early SMILe indexes of < 0.3 and > 0.7, respectively, even using late imaging (Fig. 7). When the early SMILe index fell between 0.3 and 0.7, 7% and 20% of patients respectively manifested late SMILe indexes of < 0.30 and > 0.70. Based on SMILe indexes of > 0.5 and ≤ 0.5, patients with early SMILe indexes > 0.7 and < 0.3 retained their MIBG diagnosis. The final diagnostic rate of LBD detection was 9% and 80% in groups with low (< 0.3) and high (> 0.7) early SMILe indexes, respectively.

## Discussion

Here, we proposed a new SMILe index by applying a logistic model for ^123^I-MIBG H/M ratios in consecutive patients. As this index indicated a likelihood of LBD (probability units, 0 to 1) derived from the distribution of the H/M ratio, it corresponded to the normalized numerical value for LBD. The results suggested that ~ 80% of ambulatory patients at our institution do not need another evaluation by late imaging if their early SMILe index is in the range of either < 0.3 or > 0.7. Calculating WRs is not needed for diagnostic purposes.

### Standardization of H/M ratio and^123^I-MIBG

Sympathetic nerve uptake and integrity can be assessed using ^123^I-MIBG. It is currently extensively applied to patients with suspected Lewy body-related diseases, namely PD and DLB. Its accuracy has been substantiated by numerous studies in Japan and elsewhere [3–6, 9–12]. About 70% of all ^123^I-MIBG imaging in Japan is used to assess patients with neurological concerns. Thus, imaging has played a crucial role in the clinical setting. These examinations are particularly valuable for distinguishing PD and related Parkinson syndromes, as well as differentiating DLB from AD in patients with dementia. Because a distinct reduction in cardiac ^123^I-MIBG uptake can be visualized from the early stages of LBD, ^123^I- MIBG imaging is now an established biomarker of this disease [6, 30].

However, standardization of the H/M ratio was essential to construct a diagnostic index. The H/M ratio is influenced by both the scinticamera and collimator [19, 31, 32], and it tends to yield lower values when a low-energy collimator, instead of medium energy, is used. A calibration method has been adopted in Japan to standardize the medium energy collimator condition, aiming to address variations in the H/M ratio across various facilities [19, 20]. This method was created using phantom experiments, and it is now applied in diverse institutional settings in several Asian and European countries [20–22]. The established lower limit of the H/M ratio between early and late images is 2.2 according to databases in the Japanese Society of Nuclear Medicine (JSNM). However, the delineation between normal and abnormal conditions remains debatable and might be influenced by the characteristics of involved patients [33]. Consequently, we endeavored to introduce a new index that is more intuitive and relevant to a diagnosis of LBD.

With respect to age dependence, JSNM working group normal MIBG database showed a slightly decreased activity in elderly persons, indicating a decline in H/M ratios by 0.05 and 0.07 per 10 years for early and late images [27]. Age-dependent decreases in the inferior wall have been determined by SPECT imaging, especially in men [29]. However, prognostic analysis in patients with heart failure using quantitative ^123^I-MIBG uptake did not require adjustment for patient age [34]. Considering that the H/M ratio might gradually decline with age, the significance of age might emerge in a larger patient cohort. However, adding the age factor did not enhance diagnostic accuracy, and the model only with H/M ratios could be used in clinical practice.

### Characteristics of the logistic model

The SMILe index, introduced herein, possesses distinct characteristics from the H/M ratio. This index is transformed by the logistic function from H/M ratio and is a value to reflect the likelihood of LBD. In terms of diagnostic accuracy for LBD, the result of ROC analyses found that the SMILe index and H/M ratios did not inherently differ. While a similar index can be derived from WRs, it was not more diagnostically effective than early and late images.

**Fig. 8:**
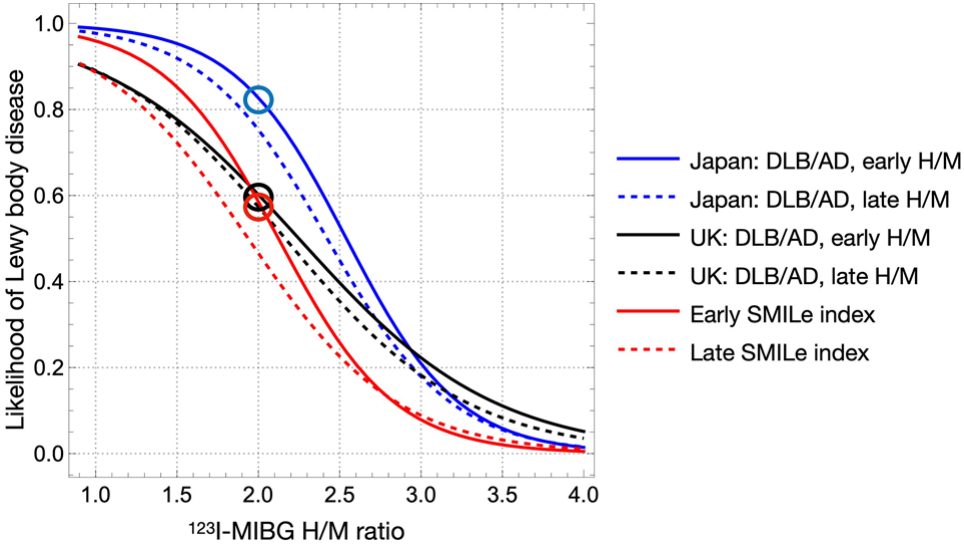
Logistic plots of (H/M) ratios and probability of LBD in three study settings. Japanese data regarding DLB and AD are from Komatsu et al. (blue) [10], and UK data from Roberts et al*.* (black) [29]. Red line, ^123^I-MIBG H/M in this study based on probability of LBD. Early and late data are shown as solid and dashed curves, respectively. Circles indicate points where early H/M ratio is 2.0. *AD* Alzheimer disease; *DLB* dementia with Lewy bodies; *H/M* heart-to-mediastinum; *LBD* Lewy body disease

A notable aspect of this index is its ability to express the probability of LBD diagnosis. When the diagnosis is clear, the logistic function forms a steeper curve in the mid-range of the H/M ratio as illustrated in Fig. 8. This is in contrast to a gentler curve when numerous ambiguous factors are included. A UK study of patients with LBD found that the optimal cut-off point differs between Japanese [12] and UK [33] databases. That study notably included patients aged > 60 years with diabetes and other risk factors to represent the typical elderly population in UK. A multicenter study found that the threshold for distinguishing DLB from AD in Japanese patients was a late H/M ratio of 2.2 compared with the UK, where the appropriate cut-off was respectively around 1.77‒1.80 and 1.61‒1.70 for early and late H/M ratios. The effects of such differences among populations have been discussed [35].

As shown in Fig. 8, the logistic curve in the mid-H/M range was steeper in the Japanese multicenter data which included only typical cases of DLB and AD. In contrast, we found that including consecutive patients with various complications in our study shifted the logistic curves leftward, suggesting a closer alignment with the UK data. Assuming an early H/M ratio of 2.0, the probability of LBD was 80% in the Japanese multicenter study, but 60% in both the UK and Japanese datasets.

Another advantage of the SMILe index is that the diagnostic performance of early and late MIBG images remains consistent and is not influenced by fixed or proportional errors between the acquisition times. Since early and late SMILe indexes are respectively normalized for the probability of LBD, both indexes were almost identical (Figs. 5B and 6B). Additionally, the SMILe index enhances diagnostic clarity by quantifying the likelihood of LBD, making it more intuitively understandable than using the H/M ratio alone. Furthermore, when the H/M ratio is diagnostically equivocal and follow-up is necessary, serial changes in LBD probability over time can be evaluated by monitoring shifts in the SMILe index.

### Selection of patients who will need late images

Both early and 3‒4 h late images are routinely acquired in many institutions. Some studies have indicated that early and late H/M ratios have comparable diagnostic accuracy [30, 36, 37]. For instance, a study of 453 patients with LBD [36] found that the overall sensitivity, specificity, and accuracy were 72.2%, 93.1%, and 84.3%, respectively, when the cut-off value was 2.2. Late H/M ratios or visual findings did not significantly differ. Accuracy results were slightly higher for delayed, than early images (73 vs. 77%) in 167 patients, but the designation changed between early and delayed images in only 8 (5%) of 167 of images [30]. Our results are essentially in agreement with these findings.

Nevertheless, many institutions still opt for both imaging evaluations to prevent diagnostic errors around potential cut-off values. One reason for this approach is the lack of a clear threshold to determine candidates for late imaging. Consequently, a SMILe index in the range of 0.3‒0.7 might offer a more practical and intuitive approach to selecting patients for whom late imaging is essential. When a SMILe index is either < 0.3 or > 0.7, late imaging would not be required. Although the proportion of patients who can forego late imaging might vary among institutions, our clinical outpatient study suggested that ≥ 80% patients might be spared having to undergo additional imaging, which might provide some comfort to individuals with LBD.

Utilizing the SMILe index developed in this study, the need for late imaging can be assessed in a Japanese multicenter study involving DLB and AD [12] (Supporting Figure). In this context, 9% of patients fell within the borderline zone of the index, ranging between 0.3 and 0.7. When the early SMILe index was < 0.3 or > 0.7, the diagnosis was not altered based on high or low MIBG imaging findings. Our results suggest that thresholds of 0.3 and 0.7 could also be applied to both PD and DLB despite the relatively small cohort of patients with DLB.

### Limitations

The primary limitation of this study is that ~ 100 patients were selected over 3 years from an outpatient setting at Kanazawa University. While a larger cohort would be preferable, the similarity of logistic curves between the UK and Japan suggests that the statistical fit for a diagnosis of LBD is acceptable. The adequacy of the model can be further assessed at diverse institutions. Due to enrolling consecutive patients, some follow-up diagnoses might have been inaccurate. A multicenter AD/DLB study found that an extended follow-up can yield more precise diagnoses, as well as their potential conversion [4, 12]. We excluded patients with young-onset PD (age < 50 years) from modeling as they have different biomarker profiles [23], and genetic PD involves pathologies not associated with α-synuclein gene, in which ^123^I-MIBG reduction depends on the type of mutation [14, 24]. Patients with amyloidosis were also excluded because the indications for using ^123^I-MIBG differ in these cases. It is crucial to consider these exclusions when applying the SMILe index in clinical studies. Furthermore, although this study is based on planar imaging, there may be a role for single-photon emission computed tomography equipped with X-ray computed tomography for absolute quantitation [38]. However, further research is needed to explore this possibility.

## Conclusion

A sympathetic ^123^I-MIBG index using a logistic model and consecutive patient data revealed the likelihood of having Lewy body-related diseases. Although the diagnostic performance was comparable with the H/M ratios, the SMILe index provided a normalized probability of LBD ranging from 0 to 1, with a cut-off at 0.5, enhancing intuitive understanding for likelihood of LDB. Additionally, indexes of either < 0.3 or > 0.7 can be used to select patients who can forego late imaging.

## Supplementary Information

Below is the link to the electronic supplementary material.

Supplementary file1 (PDF 538 KB)

## Abbreviations

AD: Alzheimer disease
AUC: Area under the curve
DLB: Dementia with Lewy bodies
H/M: Heart-to-mediastinum
LBD: Lewy body disease
MDS: Movement Disorder Society
MIBG: Metaiodobenzylguanidine
ROC: Receiver operating characteristics
SMILe index: Sympathetic MIBG Index for Lewy body disease
WR: Washout rate

## Declarations

### Data availability

The datasets generated during and/or analyzed during the current study are available from the corresponding author on reasonable request.
