# Supplementary material for: Sympathetic 123I-metaiodobenzylguanidine index for Lewy body disease: probability-based diagnosis and identifying patients exempt from late imaging
Source: Ann Nucl Med. 2024 Jun 13;38(10):814–24. doi: 10.1007/s12149-024-01950-4 (PMC11401792; doi:10.1007/s12149-024-01950-4)
Supplement: Supplementary file 1 — Supplementary file1 (PDF 538 KB) [file 12149_2024_1950_MOESM1_ESM.pdf]

[Supporting Figure (on website)]

Sympathetic ^{123}I-metaiodobenzylguanidine index for Lewy body disease: Probability-based diagnosis and identifying patients exempt from late imaging

Kenichi Nakajima, Takeshi Matsumura, Junji Komatsu, Hiroshi Wakabayashi, Kenjiro Ono, Seigo Kinuya

Kanazawa University, Kanazawa, Japan

Application of sympathetic MIBG index for Lewy body disease (SMILe) to Japanese multicenter data involving dementia with Lewy bodies and Alzheimer disease (Reference).

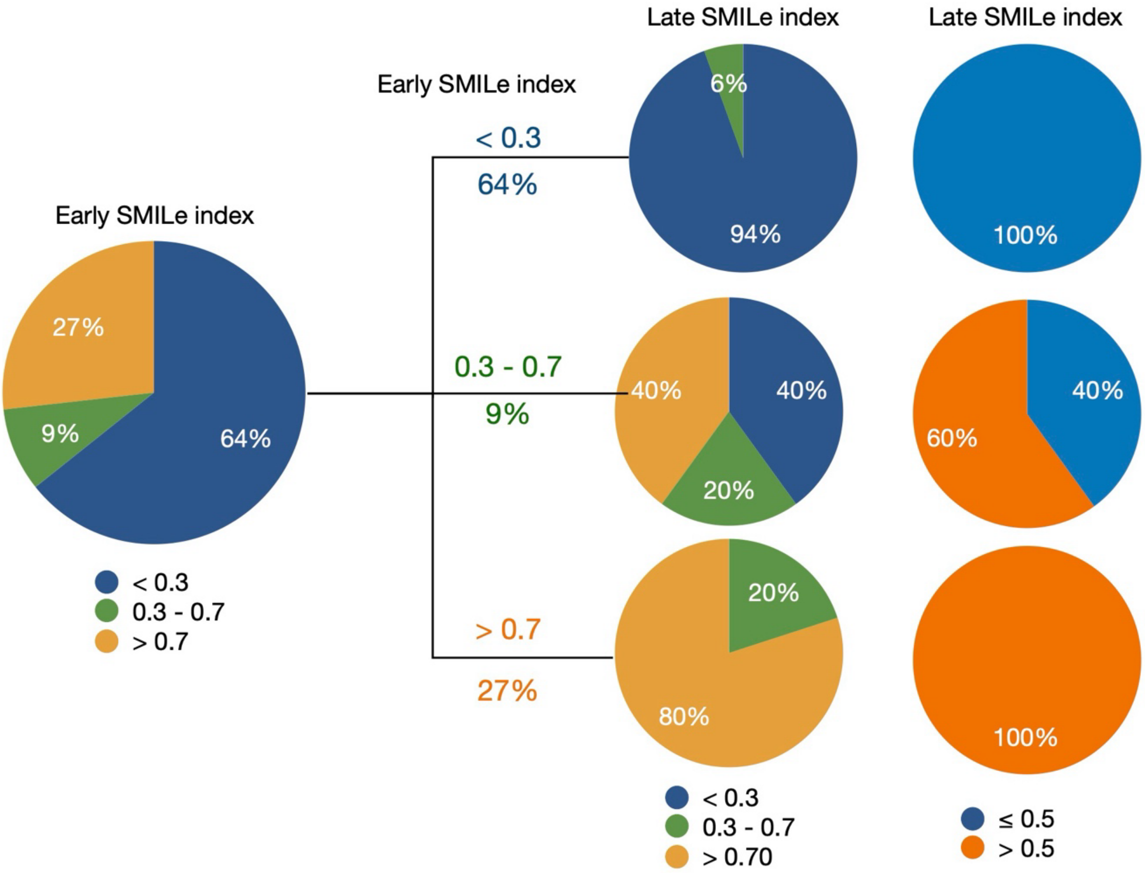

Reference:

Komatsu J, Samuraki M, Nakajima K, Arai H, Arai H, Arai T, *et al*. ^{123}I-MIBG myocardial scintigraphy for the diagnosis of DLB: a multicentre 3-year follow-up study. J Neurol Neurosurg Psychiatry. 2018; 89:1167-73
